# Acute kidney injury after in-hospital cardiac arrest in a predominant internal medicine and cardiology patient population: incidence, risk factors, and impact on survival

**DOI:** 10.1080/0886022X.2021.1956538

**Published:** 2021-07-27

**Authors:** Sammy Patyna, Kirsten Riekert, Stefan Buettner, Anna Wagner, Johannes Volk, Helge Weiler, Julia W. Erath-Honold, Helmut Geiger, Stephan Fichtlscherer, Jörg Honold

**Affiliations:** a Department of Internal Medicine III/Nephrology, University Hospital Frankfurt, Frankfurt, Germany; b Department of Internal Medicine III/Cardiology, University Hospital Frankfurt, Frankfurt, Germany

**Keywords:** In-hospital cardiac arrest, acute kidney injury, hemodialysis, prognosis

## Abstract

**Introduction:**

Prognosis of survivors from cardiac arrest is generally poor. Acute kidney injury (AKI) is a common finding in these patients. In general, AKI is well characterized as a marker of adverse outcome. In-hospital cardiac arrest (IHCA) represents a special subset of cardiac arrest scenarios with differential predisposing factors and courses after the event, compared to out-of-hospital resuscitations. Data about AKI in survivors after in-hospital cardiac arrest are scarce.

**Methods:**

In this study, we retrospectively analyzed patients after IHCA for incidence and risk factors of AKI and its prognostic impact on mortality. For inclusion in the analysis, patients had to survive at least 48 h after IHCA.

**Results:**

A total of 238 IHCA events with successful resuscitation and survival beyond 48 h after the initial event were recorded. Of those, 89.9% were patients of internal medicine, and 10.1% of patients from surgery, neurology or other departments. In 120/238 patients (50.4%), AKI was diagnosed. In 28 patients (23.3%), transient or permanent renal replacement therapy had to be initiated. Male gender, preexisting chronic kidney disease and a non-shockable first ECG rhythm during resuscitation were significantly associated with a higher incidence of AKI in IHCA-survivors. In-hospital mortality in survivors from IHCA without AKI was 29.7%, and 60.8% in patients after IHCA who developed AKI (*p* < 0.01 between groups).

By multivariate analysis, AKI after IHCA persisted as an independent predictor of in-hospital mortality (HR 3.7 (95% CI 2.14–6.33, *p* ≤ 0.01)).

**Conclusion:**

In this cohort of survivors from IHCA, AKI is a frequent finding, with adverse impact on outcome. Therefore, therapeutic strategies to prevent AKI in post-IHCA patients are warranted.

## Introduction

Each year, approximately 292,000 in-hospital cardiac arrests (IHCA) occur in US hospitals [1]. Over the past decades, the incidence of IHCA seems to increase, from about 6.5 events per 1000 hospital admissions per year (US data from 2003 to 2007) [2], to 9.5 IHCA per 1000 hospital admissions in the US from 2008 to 2017 [1,3]. Data from the UK suggest a lower incidence (1.6 IHCA per 1000 hospital admissions in the UK, data from 2011 to 2013) [4]. Prognosis after IHCA is poor, with a survival rate of 20% observed in large studies [5]. Although several therapeutic procedures like initiation of CPR, pharmacotherapy, defibrillator use and airway management can be initiated faster in an IHCA situation [6], prognosis after IHCA is poor: a recent study in more than 1,000,000 survivors from IHCA found an average one-year survival rate of 13% [7]. However, prognosis seems to improve over time [8,9].

Compared to out-of-hospital cardiac arrest (OHCA), IHCA represents a poorly investigated clinical condition. In a recent meta-analysis of 92 randomized cardiac arrest studies, only 4 (4%) included exclusively IHCA patients [10]. Compared to OHCA victims, IHCA represent a more heterogenous patients’ collective, with a higher proportion of non-cardiac-related resuscitation etiology, e.g., due to respiratory disorders. Acute kidney injury (AKI) following cardiopulmonary resuscitation is a frequent finding. Independently from preexisting chronic kidney disease, the quality of cardiopulmonary resuscitation and duration of shock after return of spontaneous circulation (ROSC) is a predisposing factor for post-resuscitation AKI [11]. One meta-analysis from 2016 described an incidence of AKI classified by common criteria like RIFLE [12] or AKIN [13] of 52% [5].

In general, AKI is associated with adverse outcome [14,15]. For survivors from out of-hospital arrest, it is shown that recovery from AKI is a good predictor of favorable neurologic outcome and hospital discharge [16].

Therefore, the aim of the present study was to describe the incidence, risk factors, and prognostic impact of acute kidney function in a contemporary cohort of survivors from in-hospital cardiac arrest.

## Methods

In this retrospective single-center study, all patients achieving ROSC after IHCA with a minimum survival period of at least 48 h were selected for analysis at Goethe University Hospital between 2006 and 2014. The identification of patients for this study was performed by electronic health record and on basis of OPS-code for cardiopulmonary resuscitation. All patients underwent resuscitation according to the guidelines of the European Resuscitation Council (ERC) [17] and were all aged above 18 years. Twelve percent of the post-resuscitation patients after IHCA with shockable first ECG rhythm received therapeutic temperature management (TTM). Follow-up data were collected from the electronic health record and include the whole hospitalization period until either discharge or death. The study was approved by the local ethics committee (reference number: 115/15) and it was conducted according to the principles of the Declaration of Helsinki.

### Classification of kidney injury

To evaluate the impairment of kidney function, the first serum creatinine at ICU within 1 h after resuscitation was used as baseline value instead of historical creatinine values, according to the European Best Practice Statement (ERBP) position statement on AKI [18]. The AKI was defined according to the AKIN classification (AKI Network) which is subdivided into three stages (AKIN stages 1–3) [13]. AKIN stage 1 is specified as increase of serum creatinine of >0.3 mg/dl or the 1.5 to 1.9-fold increase of serum creatinine by comparison to baseline within 48 h or urine output less than 0.5 m/kg per hour for more than 6 h [12]. Stage 2 is defined as increase of serum creatinine of 2–2.9-fold within 48 h or urine output less than 0.5 m/kg per hour for more than 12 h [12]. In stage 3, an increase of serum creatinine >4 mg/dl with an urgent rise of >0.5 mg/dl or an increase of serum creatinine of more than 300% compared to baseline creatinine or urine output less than 0.3 m/kg per hour for 24 or anuria for 12 h occurs [12].

### Statistical analysis

Statistical analysis was performed in BiAS (v11.01; Epsilon-Verlag, Nordhasted, Germany). Continuous variables are shown as median and interquartile range (IQR). For multiple group comparisons, statistical significance was stated performing one-way ANOVA followed by Bonferroni’s *post hoc* correction or Kruskal–Wallis test followed by Dunn’s *post hoc* correction for parametric and nonparametric data, respectively. Normality was assessed by the Kolmogorov–Smirnov test. Spearman’s rank correlation analysis was used to determine bivariate relationships by calculating Spearman’s Rho (correlation coefficient). To evaluate the determinants of independent predictors of AKI, univariate and multivariate logistic regression analyses were performed and odds ratio (OR) and corresponding 95% confidence interval were calculated. Kaplan–Meier estimator and corresponding log-rank test was used for survival analysis. A two-sided *p* value of <0.05 was considered as statistically significant.

## Results

**Figure 1.**
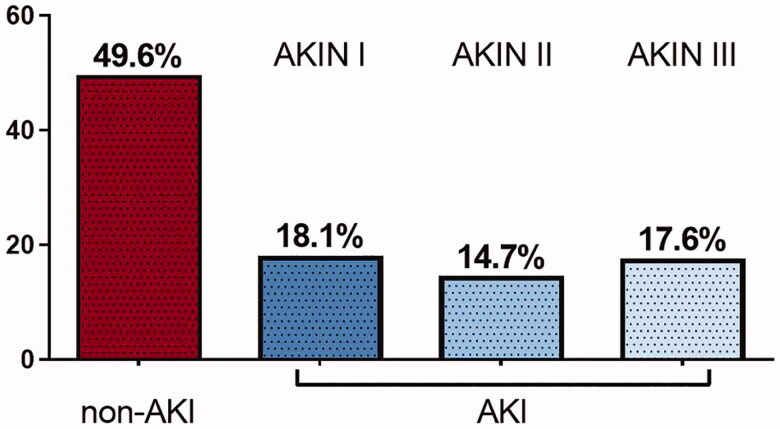
Percentage of AKI-incidence after IHCA, subdivided into AKIN-classification stadium 1–3. AKIN: acute kidney injury network.

A total number of 238 survivors from IHCA and survival of 48 h and beyond were included in this analysis. Of those, 120 patients (50.4%) developed AKI as defined by the AKIN classification [12]. The majority of AKI patients were classified as AKIN stages 1 and 2 (32.8% of all patients), only 17.6% of the patients developed AKIN stage 3 (see Figure 1). In 28 patients (23.3% of the AKI group), acute renal replacement therapy had to be initiated. Fifteen patients (12.5% of the AKI group) were on chronic renal replacement therapy prior to IHCA.

Table 1 depicts the baseline characteristics of these patients and differences between the AKI- and non-AKI-groups. Patients of the AKI-group were significantly older (median age 73 years, IQR 64–81 years versus 68 years, IQR 56–78 years, *p* = 0.03); with a 100% longer duration of resuscitation period compared to the non-AKI-group (20 min, IQR 5–40 min versus 10 min, IQR 3–20 min, *p* < 0.01). Consecutively, first pH and lactate levels at admission to ICU had deteriorated more significantly in the AKI-group (pH: 7.21, IQR 7.09–7.34 versus 7.28, IQR 7.16–7.39, *p* = 0.01; lactate: 52.52 mg/dl, IQR 25.24–101.22 mg/dl versus 40 mg/dl, IQR 19.9–71.1 mg/dl, *p* = 0.02) with higher potassium levels in the AKI-group (4.3 mmol/l, IQR 3.7–5 mmol/l) than in the non-AKI-group (4.0, IQR 3.6–4.6 mmol/l, *p* = 0.03). Blood gas measurements of oxygenation and CO_2_ elimination did not differ significantly between groups; however, a trend toward hyperoxygenation with 28% higher arterial pO_2_ levels in the AKI-group could be detected (pO_2_ at admission: 148.5 mmHg, IQR 83.3–289.2 mmHg versus 116 mmHg in the non-AKI-group, IQR 78.6–268.5 mmHg).

**Table 1.** Basic patient characteristics.

	AKI	Non-AKI	*p* Value
Parameter	*n* = 120	*n* = 118	
Baseline characteristics			
Age [years]	73 (64–81)	68 (56–78)	0.033
Gender (male) [% (*n*)]	72 (86)	67 (79)	0.483
Mortality [% (*n*)]	60 (73)	30 (35)	<0.001
Duration of CPR [minutes]	20 (5–40)	10 (3–20)	<0.001
Initial creatinine [mg/dl]	1.56 (1.20–2.22)	1.23 (0.90–1.92)	<0.001
Creatinine at discharge [mg/dl]	1.34 (0.76–1.77)	0.85 (0.67–1.05)	<0.001
1st shockable Rhythm [% (*n*)]	22 (26)	36 (43)	0.015
Duration of hospitalization [days]	17 (8–31)	19 (10–31.5)	0.340
Mechanical ventilation at IMC [days]	6 (3–13)	6 (2–11)	0.110
Comorbidities			
Chronic kidney disease [% (*n*)]	46 (55)	42 (49)	0.516
Coronary heart disease [% (*n*)]	61 (73)	63 (74)	0.791
Arterial hypertension [% (*n*)]	79 (95)	72 (85)	0.228
Diabetes mellitus [% (*n*)]	39 (47)	38 (45)	0.895

Data: median (IQR); % (*n*). AKI: acute kidney injury; CPR: cardiopulmonary resuscitation; IMC: intermediate care.

Baseline creatinine was higher in the group with consecutive AKI (median 1.56 mg/dl, IQR 1.2–1.22 mg/dl) than among patients with preserved kidney function after IHCA (1.23 mg/dl, IQR 0.9–1.92 mg/dl, *p* < 0.01).

The vast majority of IHCA in this study population originate from the department of internal medicine (89.9%), presumed causes of IHCA were predominantly cardiac related (44.9%, see Table 2).

**Table 2.** Causes of IHCA.

	All	*n* = 238	AKI	*n* = 120	Non-AKI	*n* = 118	*p* Value
		%	(*n*)	%	(*n*)	%	(*n*)	
Referral from internal medicine	89.9	(214/238)	94.3	(113/120)	85.6	(101/118)	0.0320
Cardiac causes							
Coronary angiography/PCI	15.9	(34/214)	19.5	(22/113)	11.9	(12/101)	0.1389
Cardiac arrhythmia	10.8	(23/214)	3.5	(4/113)	18.8	(19/101)	0.0001
Myocardial infarction	6.1	(13/214)	3.5	(4/113)	8.9	(9/101)	0.1506
Course after TAVI	5.6	(12/214)	6.2	(7/113)	5.0	(5/101)	0.7724
Implantation of pacemaker/ICD	2.3	(5/214)	2.7	(3/113)	2.0	(2/101)	1.0000
Transcatheter mitral valve repair	0.5	(1/214)	0.0	(0/113)	1.0	(1/101)	0.4719
Severe electrolyte imbalance	1.9	(4/214)	2.7	(3/113)	1.0	(1/101)	0.6239
Pulmonary embolism	1.9	(4/214)	3.5	(4/113)	0.0	(0/101)	0.1238
Non-cardiac causes							
Event on intermediate care unit	7.5	(16/214)	9.7	(11/113)	5.0	(5/101)	0.2046
Aspiration	1.4	(3/214)	0.9	(1/113)	2.0	(2/101)	0.6032
Oncological patient	1.9	(4/214)	1.8	(2/113)	2.0	(2/101)	1.0000
Sedation and intubation	1.9	(4/214)	2.7	(3/113)	1.0	(1/101)	0.6239
Sepsis	0.9	(2/214)	1.8	(2/113)	0.0	(0/101)	0.4992
Hemodialysis	0.9	(2/214)	1.8	(2/113)	0.0	(0/101)	0.4992
Other	40.7	(87/214)	39.8	(45/113)	41.6	(42/101)	0.8892
Referral from others	8.4	(20/238)	3.3	(4/120)	13.6	(16/118)	0.0048
Course after surgery	60.0	(12/20)	75.0	(3/4)	56.3	(9/16)	0.6186
Neurologic disorder (seizure, stroke)	30.0	(6/20)	25.0	(1/4)	31.3	(5/16)	1.0000
Other	10.0	(2/20)	0.0	(0/4)	12.5	(2/16)	1.0000
Undetermined	1.7	(4/238)	2.5	(3/120)	0.9	(1/118)	0.6219

Data: % (*n*). PCI: percutaneous coronary intervention; TAVI: transcatheter aortic valve replacement; ICD: implantable cardioverter defibrillator.

The incidence of comorbidities such as coronary artery disease, arterial hypertension, or diabetes mellitus did not differ significantly between patients of the AKI- and non-AKI-groups (see Table 1). The first ECG recording during resuscitation documented a shockable rhythm in 43 patients (36%) of the patients with preserved kidney function after resuscitation. In contrast, only 26 patients of the AKI-group (22%) had a shockable first rhythm on ECG during resuscitation, *p* = 0.015 between groups.

*AKI and in-hospital all-cause mortality*: A total of 108/238 survivors from IHCA died during the further in-hospital course, accounting for an in-hospital all-cause mortality of 45.4%. Of the 120 patients who developed AKI after IHCA, 73 patients deceased during further in-hospital course (60.8%), whereas the mortality was 29.7% in the subgroup of patients with preserved kidney function after IHCA (*p* < 0.01).

Comparing the subgroup of internal medicine patients with cardiac causes for IHCA to patients without cardiac causes, the incidence of AKI did not differ significantly, with a trend toward higher rates of AKI in patients with non-cardiac causes for IHCA (52.8% versus 49.5%, *p* = 0.411). Moreover, mortality rate after AKI was non-significantly higher in patients with non-cardiac-related IHCA (63.1 versus 54.2%, *p* = 0.439). Patients with cardiac-related IHCA and preserved kidney function had a significantly lower in-hospital mortality rate, compared to that of non-cardiac related IHCA patients without AKI (14.3% versus 40.4%; *p* = 0.046). In internal medicine patients with cardiac-related IHCA, the occurrence of AKI was associated with a 3.7-fold increase of in-hospital mortality, whereas AKI in internal medicine patients without cardiac-related IHCA only was associated with only a 1.56-fold increase of mortality.

**Figure 2.**
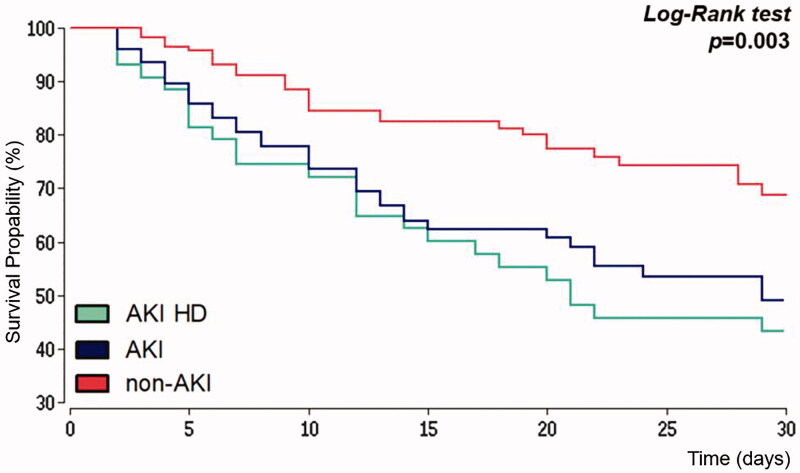
Kaplan–Meier estimator. Survival within 30 days after in-hospital cardiac arrest. AKI: acute kidney injury; HD: hemodialysis.

Figure 2 represents Kaplan–Meier analysis for all-cause mortality within 30 days after IHCA for patients with preserved kidney function, patients with AKI after IHCA and those AKI-patients with new onset of renal replacement therapy. The median survival time for patients without AKI was 39 days and 29 days in the AKI-group. The probability of survival day 30 after IHCA was 69% in patients with preserved kidney function and 47% in the AKI-group. For AKI-patients with new-onset of renal replacement therapy, the probability of 30 days survival was even more reduced to 41%. Hazard ratio for all-cause mortality 30 days after IHCA was 2.04 (95% CI 1.37–3.02) when AKI was observed.

**Figure 3.**
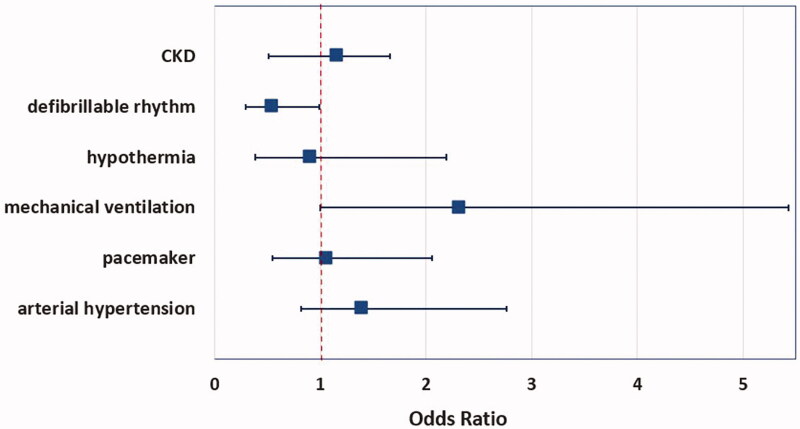
Forest plot. Multivariate analysis of AKI comorbidities after IHCA. CKD: chronic kidney disease.

By multivariate analysis, only a non-shockable rhythm at first ECG documentation persisted as an independent predictor of AKI in these patients (Figure 3).

## Discussion

Data about AKI after successful IHCA in contemporary patients are rare. Due to the heterogeneity of IHCA scenarios, estimation of prognosis after survival remains a complex task. Moreover, it appears questionable whether AKI features from OHCA survivors can be applied to IHCA victims beyond doubt.

Our findings in a cohort of internal medicine patients who survived IHCA are consistent with previous studies on cardiac arrest victims [5], describing a high incidence of early AKI with 50.4% and comparable adverse prognostic impact of AKI in IHCA survivors with an almost doubled in-hospital mortality rate for the whole study population. Internal medicine patients without cardiac-related causes for IHCA show a 1.5-fold increase of mortality and patients with cardiac causes for IHCA present with a 3.7-fold mortality rate after developing AKI. These findings reveal that patients after IHCA caused by cardiac events are at the same risk to develop AKI, but apparently are much more vulnerable to AKI.

As shown before in other studies, our data reveal that age, duration of CPR, and a shockable first rhythm on ECG during resuscitation had an influence on the incidence of AKI in this study population. However, only a non-shockable rhythm at first ECG documentation could be confirmed as an independent predictor of AKI. The observation of lower rates of new onset renal replacement therapy in our study with 23% versus 33% RRT in the review of Sandroni et al. did not translate in reduced mortality, with a hazard ratio for in-hospital mortality of 1.66 (95% CI 1.45–2.63) [5], contradictory to our findings as AKI doubled the risk for in-hospital mortality. As in OHCA survivors, younger patients’ age and the first ECG rhythm was indicative for a lower probability of AKI in the post-arrest course if a shockable rhythm (ventricular tachycardia or ventricular fibrillation) was detected. This finding can be explained by a probably shorter duration of shock with less pronounced post-cardiac arrest syndrome [19,20] due to shorter no flow time, regardless whether bystander-CPR was performed or not [21].

However, we cannot provide more detailed data about the mechanism of death in our AKI group after survival of IHCA. As the early mortality after cardiac arrest is mainly driven by cardiac causes [22,23], further investigation of the interaction between AKI and cardiac failure or neurological outcome would be of interest. Due to the heterogeneity of patients in this study from internal medicine and other disciplines, different patient-related and IHCA-related factors as well as different post-resuscitation therapy, our findings might not be applicable on every IHCA collective.

Another important limitation of this study is the definition of AKI in the context of cardiac arrest, as performed with the AKIN classification in this study. The time frame for diagnosis of AKI might appear arbitrary but aims to comprise the immediate association of shock and reperfusion injury due to cardiac arrest and development of AKI, which could also be shown before in experimental studies [21]. However, we cannot rule out that another definition than the AKIN classification [24] would have influenced these findings. Nevertheless, the prognostic power of AKIN-classification for in-hospital mortality is comparable to AKI diagnosed by KDIGO-criteria in critical ill patients [22].

Due to the retrospective nature of the study, we are unable to provide more detailed insight into post cardiac arrest therapy and a possible impact of preventive strategies on the development of AKI and outcome. The CASPRI score from 2012 aimed to provide prognostic information based on patients’ baseline characteristics, comorbidities and resuscitation quality issues and does not comprise factors from the post-resuscitation period [25]. We only can speculate whether integration of new-onset AKI as defined in our study improves the score’s prognostic utility.

Current guidelines recommend targeted temperature management (TTM) for post-resuscitation patients after out-of-hospital cardiac arrest, with less evidence for IHCA patients [18]. One recent study postulated that RRT in TTM is independently associated with a lower risk of death after OHCA [26]. In the present study, only 28 patients (12%) of our study population were assigned to TTM. The lower incidence of AKI under TTM (46% versus 51% without TTM, OR 0.91, 95% CI 0.38–2.19) did not translate into reduced mortality (61% versus 43%), as one study provides conflicting results about the association of TTM and AKI [27,28]. Further studies are warranted to investigate the causal relationship between TTM and AKI in the IHCA setting.

## Conclusion

Regardless of the different peri-cardiac arrest conditions, both the incidence and the prognostic impact of AKI in IHCA survivors are enormous and comparable to that described in OHCA victims [29]. Fast and effective onset of resuscitation is suggested to be a cornerstone of AKI prevention in order to improve prognosis in these seriously ill patients’ population. Whether specific post-resuscitation therapy regimens can reduce incidence of AKI in these patients has to be investigated in further studies.
